# Case Report: Incidental finding of Zinner syndrome in an asymptomatic 53-year-old Palestinian male

**DOI:** 10.3389/fruro.2023.1233897

**Published:** 2023-07-26

**Authors:** Lila H. Abu-Hilal, Duha I. Barghouthi, Yumna Njoum, Amal Obeid, Khaled Alshawwa, Tawfiq AbuKeshek, Mohammed Maree

**Affiliations:** ^1^ Faculty of Medicine, Al-Quds University, Jerusalem, Palestine; ^2^ Department of Surgery, Al-Makassed Hospital, Jerusalem, Palestine; ^3^ Department of Radiology, Al-Makassed Hospital, Jerusalem, Palestine

**Keywords:** Zinner syndrome, seminal vesicle cyst, ejaculatory duct obstruction, ipsilateral renal agenesis, incidental CT finding, double inferior vena cava, high aortic bifurcation, case report

## Abstract

**Introduction:**

Zinner syndrome (ZS) is a rare condition characterized by a triad of seminal vesicle cyst (SVC), ipsilateral ejaculatory duct obstruction, and ipsilateral renal agenesis. The diagnosis is often delayed due to non-specific symptoms, such as lower urinary tract symptoms and infertility, typically appearing in the second and third decades of life.

**Case presentation:**

We present the first published case of ZS in Palestine, involving a 53-year-old male patient who sought medical attention for right-sided hernia repair. Pre-operative imaging revealed a combination of findings, including a solitary left kidney with cysts, mild hydronephrosis, an enlarged prostate, suspicious soft tissue density, and abnormal lymph nodes. The diagnosis of ZS was confirmed through an abdominal ultrasound, identifying a dilated seminal vesicle and completing the criteria of ZS.

**Discussion:**

The typical for ZS is to present in late second decade of life with nonspecific urogenital symptoms and infertility, However, our patient’s incidental diagnosis during the preoperative evaluation of incisional hernia in a relatively old age with no previous complaints, the identification of a high aortic bifurcation at the level of the left kidney and a double Inferior Vena Cava (IVC) in this case of ZS represents novel and distinctive findings not commonly reported in previous cases.

**Conclusion:**

Our patient’s presentation and findings expand our understanding of the anatomical variations associated with ZS. This case report contributes to the advancement of knowledge in the field of ZS and provides valuable insights for future clinical management and research investigations.

## Introduction

Zinner Syndrome, a rare condition first reported in 1914, is characterized by a unique triad of seminal vesicle cyst, ipsilateral ejaculatory duct obstruction, and ipsilateral renal agenesis (1).

The syndrome arises from insults during embryogenesis of the mesonephric duct in males, leading to its development (2). Diagnosis of ZS is often delayed due to the presence of non-specific symptoms, including lower urinary tract symptoms e.g. dysuria, urgency, hematuria, perineal pain or flank pain and infertility consisting of 45% of cases, during the second and third decades of life. The reported incidence of this syndrome was as low as 0.0021% and the mean age at diagnosis was 29.35 years (3, 4).

Notably, this male-specific disorder shares similarities with the female Mayer-Rokitansky-Kuster-Hauser syndrome (2). In this case report, we present the incidental discovery of ZS in a 53-year-old patient who sought medical attention for hernia repair at our hospital. 214 cases have been reported in the literature from 1999 to 2020 (3). To our knowledge, this is the first published case of ZS in Palestine.

## Case presentation

We present the case of a 53-year-old male patient with a medical history significant for diabetes mellitus, hypertension, congenital left single kidney, chronic hepatitis C infection, known penicillin and contrast allergy, and previous open cholecystectomy. The patient was admitted for elective right-sided incisional hernia repair with mesh following previous open cholecystectomy that was done 6 months ago.

Prior to the scheduled surgery, an abdominal and pelvic computed tomography (CT) scan without intravenous (IV) contrast was performed, interestingly revealing several notable findings. The scan confirmed the presence of an 11.7 cm single left kidney which exhibited two cysts, the largest measuring 3.3 cm, accompanied by a small non-obstructive renal stone. Additionally, minimal left-sided hydroureteronephrosis was observed, and no ureteric stones were detected. The prostate was found to be enlarged, estimated at a volume of 54 cc.

**Figure 1:**
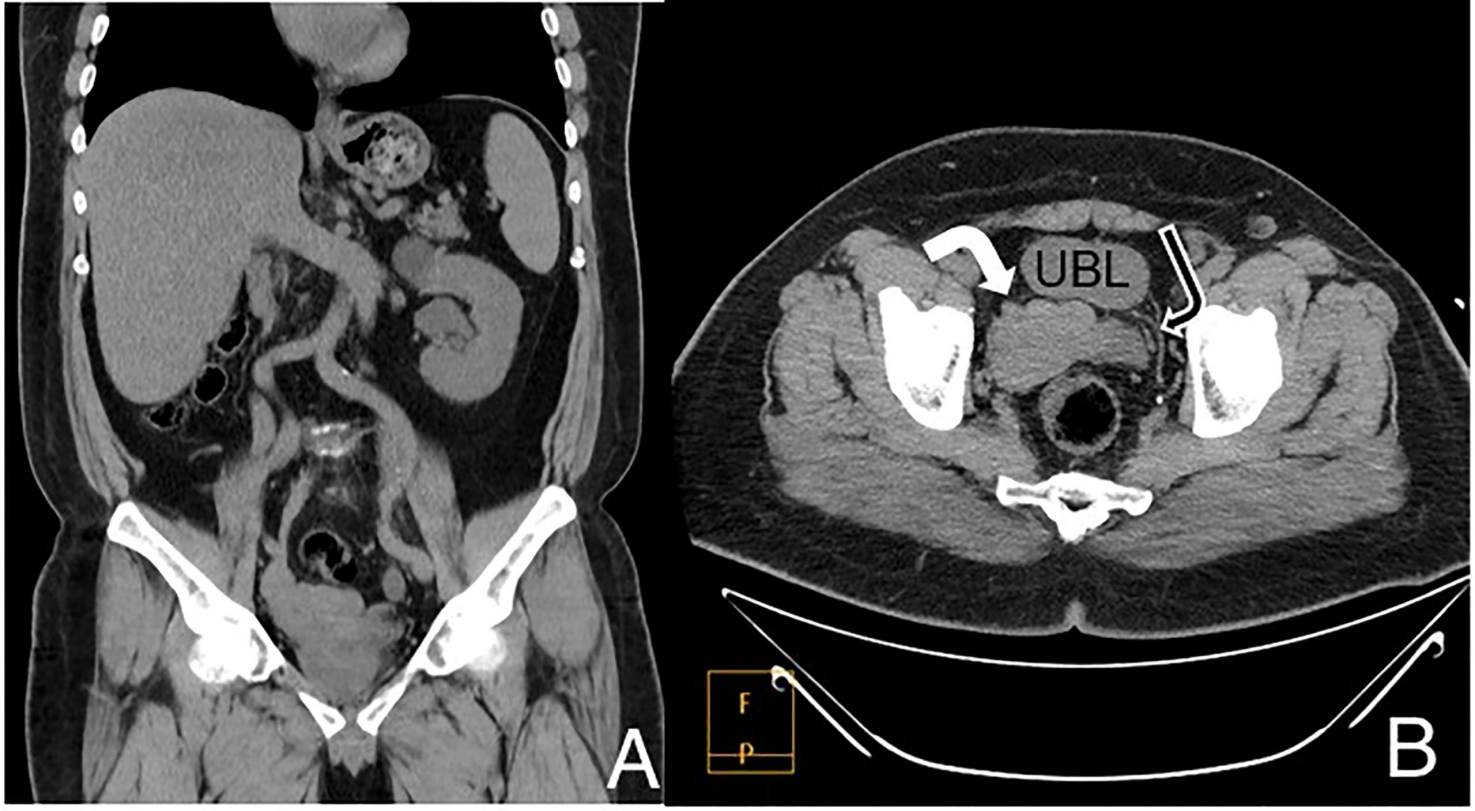
CT scan without IV contrast coronal **(A)** and axial **(B)** images. The upper abdomen shows a single left kidney containing a simple upper pole cyst with absent right kidney. Images in the pelvis showed a lobulated process replacing the right seminal vesicle (white arrow), normal left side for comparison (black arrow). UBL, Urinary bladder.

**Figure 2:**
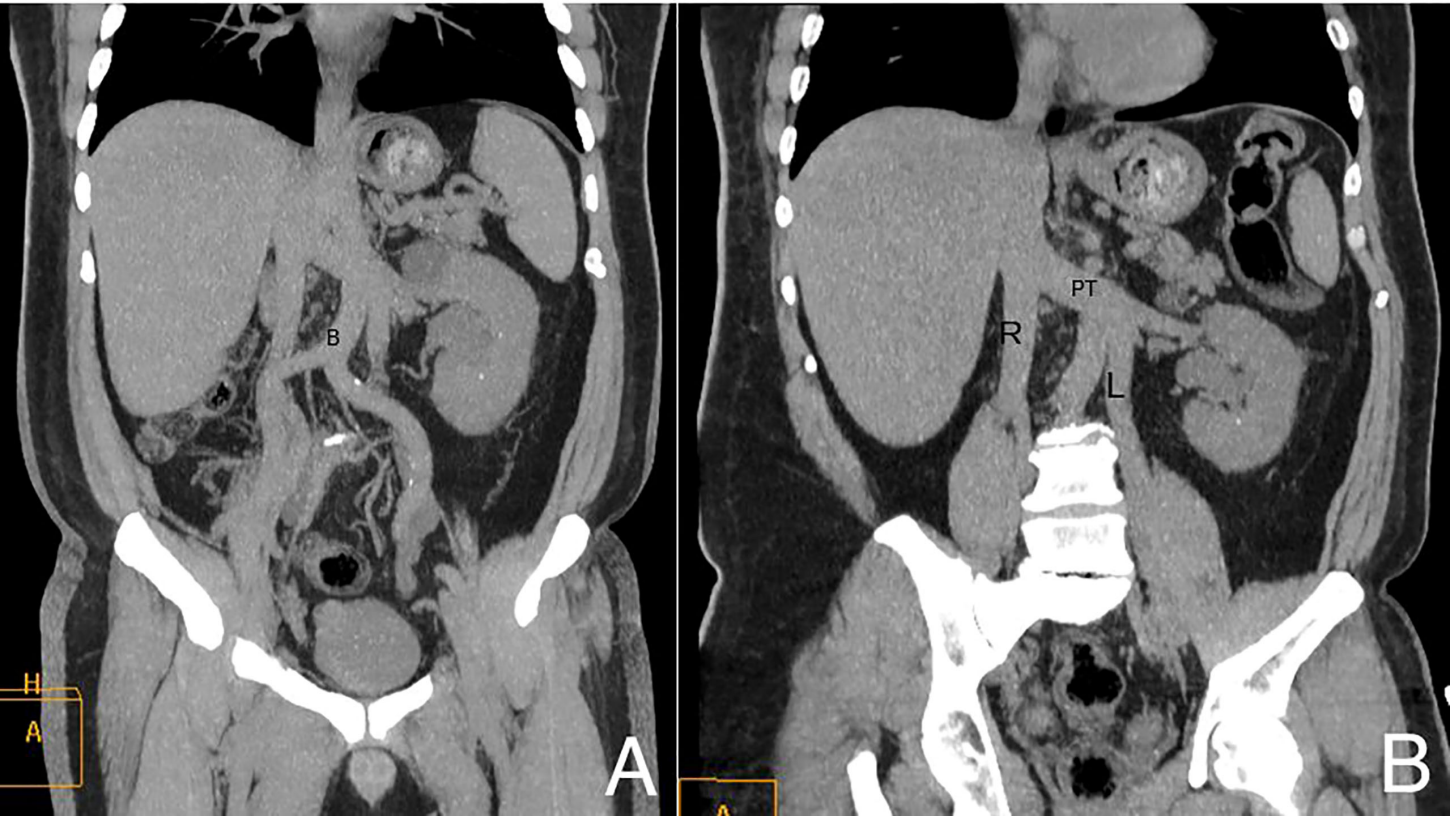
Coronal Maximum Intensity Projection (MIP) **(A)** and coronal oblique **(B)** CT images show the high bifurcation (B in the image) of the aortic arch and double IVC. R, right inferior vena cava; L, left inferior vena cava; PT, preaortic trunk.

**Figure 3:**
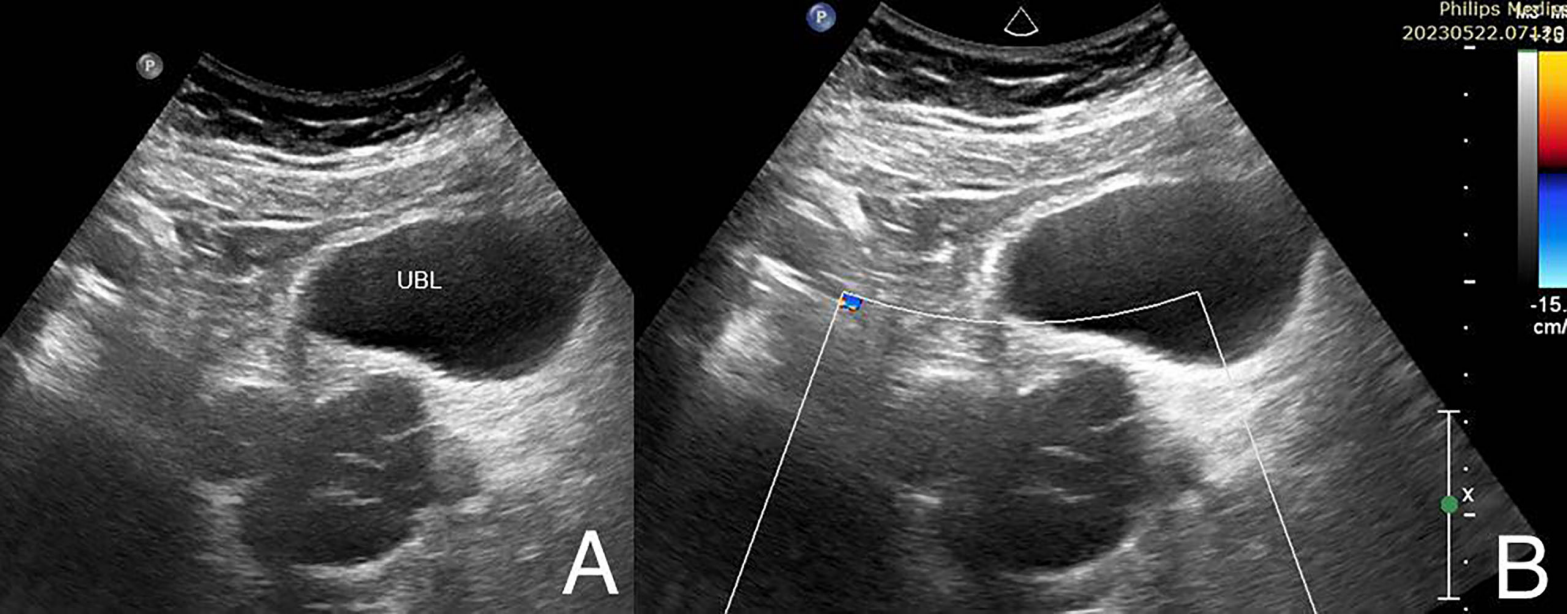
Right paramedian sagittal gray scale **(A)** and color Doppler **(B)** US images show lobulated septated predominantly anechoic cystic process posterior to the urinary bladder (UBL) representing the dilated right seminal vesicle. No internal vascularity on color Doppler interrogation.

Intriguingly, a lobulated process with soft tissue-like density measuring approximately 4 cm with a Hounsfield unit value of 40, was observed, occupying the anatomical location of the right seminal vesicle and inseparable from the right upper surface of the prostate, necessitating further assessment **(** **Figure 1** **)**. A high aortic bifurcation at the level of the left kidney (L3 vertebral level) and a double Inferior Vena Cava were also identified (**Figure 2**). Furthermore, a large right upper quadrant hernia containing fat and small bowel loops, with a hernial defect measuring 7 cm, was observed. To further assess the urogenital findings on CT, an abdominal ultrasound was performed, confirming the right pelvic lobulated process to be a dilated seminal vesicle **(** **Figure 3** **)** and the diagnosis of ZS was confirmed, meeting the full criteria for ZS without the aid of Magnetic Resonance Imagining (MRI) to confirm the diagnosis.

Furthermore, the patient is married with 4 offspring and denies urinary symptoms except for mild dribbling attributed to benign prostatic enlargement. He has no history of dysuria, hematuria, or sexual dysfunction and denies previous recurrent urinary tract infections. No previous history of recurrent urinary tract infections. Notably, the patient remained asymptomatic despite the incidental discovery of ZS., it’s worth mentioning that semen analysis was done recently to assess fertility and results were as follows, semen volume: 6.56 ml (normal > 1.5 ml), semen pH: Alkaline (normal > 7.2), and total sperm count: 871.71mill (normal> 40mill).

In summary, this case highlights the incidental discovery of ZS in a 53-year-old male patient who presented to our hospital for incisional hernia repair. Pre-operative imaging revealed a constellation of findings, including a solitary left kidney with cysts, mild hydronephrosis, an enlarged prostate, suspicious soft tissue density, and abnormal lymph nodes.

## Discussion

ZS is a urogenital tract developmental anomaly that arises from malformation of the mesonephric duct during embryogenesis, the defining features of this syndrome include renal agenesis with ipsilateral seminal vesicle cyst and ipsilateral ejaculatory duct obstruction (2).

The age range of diagnosis of ZS varies. Typically identified during adulthood exhibiting symptoms that are vague and primarily include discomfort, such as dysuria, pollakisuria, perineal pain, epididymitis, and pain following ejaculation that usually appears upon sexual activity due to ejaculatory duct obstruction leading to accumulation of seminal fluid. However, in some cases it is possible to diagnose ZS prenatally during an ultrasound revealing unilateral renal agenesis that raises suspicion of ZS and can be confirmed *via* MRI. While, in our case, the patient presented at the relatively late age of 53-years-old and had no prior complaints related to the condition as he reported normal sexual activity and has 4 healthy children and normal semen analysis.

In the diagnosis of ZS, the combined use of ultrasound and CT urography plays a pivotal role. Careful evaluation of the imaging studies is crucial to ensure accurate diagnosis and prevent potential misdiagnoses and plan surgical intervention. This approach enables comprehensive assessment and characterization of the genitourinary abnormalities associated with ZS (5). Diagnosing asymptomatic cases is challenging, diagnostics are typically saved for patients with symptoms. Transrectal ultrasonography (TRUS) is the method most frequently employed to evaluate seminal vesicle cysts in adults as it is considered to be more accurate than transabdominal pelvic ultrasound (PUS). However, PUS scans are usually performed for the initial evaluation in these situations because it is considered less invasive. PUS usually reveals a cystic lesion in the retrovesical space and the ipsilateral kidney is absent (6). Since ultrasonography is affordable, accessible, noninvasive, and simple to perform by physicians, it is also regarded as the imaging modality of choice for follow-up in patients with asymptomatic ZS.

Up to 45% of patients may be experiencing infertility and develop abnormal semen parameters, considering surgery is exclusive for individuals who are symptomatic, the primary goals of surgical care for ZS patients are to reduce discomfort and protect the contralateral ejaculatory duct to maintain fertility (7, 8).

Surgical approaches include, open exploration with vesiculectomy, cyst aspiration transperitoneally or transrectally, and cyst deroofing transurethrally used to be common forms of treatment. Since simple aspiration of SVC has been shown to be complicated by cyst recollection infection. Pelvic exploration, aspiration of the cystic fluid, and open surgical excision of the seminal vesicle cyst combined, can be considered the ultimate therapy. Although, the open approach is being widely replaced by minimally invasive procedures including laparoscopy or *via* using robot assisted techniques, which is less invasive than standard open surgery because of the deep position of seminal vesicles in the retrovesical area (9, 10).

Despite the patient’s lack of symptoms, this case report adds to the existing medical literature by presenting an unusual age of diagnosis, highlighting the importance of incidental findings, describing unique imaging findings, and expanding the geographical representation of ZS cases. It contributes to the collective understanding of this rare condition, aiding in its recognition and management in clinical practice.

## Conclusion

In conclusion, our case report highlights the unique features and clinical presentation of ZS, a rare developmental anomaly of the urogenital tract. While this syndrome symptoms typically manifests after encountering sexual activity at puberty, our case presents an atypical scenario of delayed diagnosis in a patient who remained asymptomatic throughout. Additionally, our report introduces novel findings of a high aortic bifurcation at the level of the left kidney and a double Inferior Vena Cava, which expands our understanding of associated anatomical variations in ZS. These findings underscore the importance of comprehensive imaging evaluations and contribute to the body of knowledge surrounding this rare condition.

## Learning objectives

1. Describe the clinical presentation and diagnostic workup of Zinner syndrome in a 53-year-old male patient, discuss the rarity of Zinner syndrome and its association with unilateral renal agenesis, seminal vesicle cysts, and ejaculatory duct obstruction.
2. Mention the management options available for Zinner syndrome, including surgical intervention and follow-up care.
3. Emphasize the significance of multidisciplinary collaboration between urologists, radiologists, and reproductive specialists in the diagnosis and management of Zinner syndrome.
4. Provide an illustrative case that contributes to the existing literature on Zinner syndrome and expands our understanding of this rare urogenital anomaly and enhance awareness among healthcare professionals about Zinner syndrome to improve early detection, appropriate management, and patient outcomes.

## Data availability statement

The original contributions presented in the study are included in the article/supplementary material. Further inquiries can be directed to the corresponding author.

## Ethics statement

This case report was approved by the Ethics Committee al Al-Makassed Hospital. Informed consent was obtained from the patient prior to publication of this report. The patient’s privacy and confidentiality have been protected. Written informed consent was obtained from the individual for the publication of any potentially identifiable images or data included in this article.

## Author contributions

LA-H and DB: Literature review and manuscript preparation. YN and MM: Manuscript review and editing. KA and AO revision process and patient follow up. TA: Radiology description and figures + diagnosis part. All authors contributed to the article and submitted and approved the submitted section.
